# Prevalence and Risk Factors of Hypotension in Patients Undergoing Caesarean Section with Spinal Anaesthesia at Muhimbili National Hospital

**DOI:** 10.24248/eahrj.v9i1.837

**Published:** 2025-09-30

**Authors:** Willbroad Kyejo, Sunil Samji, Allyzain Ismal, Edwin Lugazia

**Affiliations:** a Department of Family Medicine, Aga Khan University Medical College, Dar Es Salaam, Tanzania; b Department of Anesthesia, Aga Khan Hospital, Dar Es Salaam, Tanzania; c Department of Surgery, Aga Khan University Medical College, Dar Es Salaam, Tanzania; d Department of Anesthesia, Muhimbili University of Health, and Allied Sciences, Dar es Salaam, Tanzania

## Abstract

**Background:**

Spinal anaesthesia is a common regional technique for caesarean sections, but is associated with hypotension in up to 80% of patients. Preventive measures include; intravenous fluid preloading, left uterine displacement, compression stockings, and vasopressors. This study aimed to determine the prevalence and risk factors of hypotension during spinal anaesthesia in pregnant patients undergoing caesarean section at Muhimbili National hospital.

**Methods:**

A descriptive cross-sectional study was conducted at Muhimbili National Hospital's Obstetric theatre, involving patients who received spinal anaesthesia during caesarean section from August 2021 to January 2022. The study excluded patients with sedation, anti-hypertensive, pregnancy-induced hypertension, modified Bromage score, or combination anaesthesia. Data was collected, and analysed using SPSS version 20.

**Results:**

A total of 300 patients were enrolled (calculated sample size 270 plus 10% margin). Of these, 33.3% underwent elective caesarean section and 66.7% emergency caesarean section. Most patients (92%) received 0.5% hyperbaric bupivacaine, while 8% received 5% heavy lidocaine. Hypotension occurred in 56.7% of patients (95% CI: 0.511–0.623). Risk factors included preload <10 mL/kg, higher sensory block levels, and absence of wedge positioning.

**Conclusion:**

Hypotension during spinal anaesthesia for caesarean section is common. Preventive measures, including adequate fluid preload, wedge positioning, and careful monitoring of sensory block height, are essential to improve maternal hemodynamic stability.

## BACKGROUND

Spinal anaesthesia, also known as subarachnoid anaesthesia, is a regional anaesthesia technique used to provide numbness and pain relief for surgical procedures.^1,2^ It involves the injection of a local anaesthetic medication into the subarachnoid space, which is the space surrounding the spinal cord and filled with cerebrospinal fluid.^1,2^

The local anaesthetic medication used in spinal anaesthesia blocks the transmission of nerve impulses, resulting in loss of sensation and muscle relaxation in the lower part of the body.^2^ Spread of the anaesthetic agent depends on baricity of solution, patient position, dosage, site of injection, physiological status and height, and once injected, will spread due to gravity and currents of cerebral spinal fluid flow.^3^ As the block progresses, autonomic fibers are blocked first, followed by sensory loss to touch/pinprick, then loss of proprioception, and finally motor function loss. This sequence allows for pain-free surgery or other procedures below the level of the injection.

The extent and duration of both sensory and motor blockade can be controlled by adjusting the type and dose of anaesthetic medication administered.^2^

Spinal anaesthesia is a widely used technique for caesarean section delivery due to its fast and effective sensory and motor block.^4^. It offers significant advantages in this setting, providing effective pain relief and ensuring a smooth surgical experience for the mother. One of the key advantages of spinal anaesthesia is its quick onset of action, providing rapid pain relief within minutes of administration. This allows for prompt initiation of the surgical procedure, minimising any delays or discomfort for the mother. However, one of the most common complications associated with spinal anaesthesia is hypotension, which can have adverse effects on both the mother and the foetus.^5–7^

Hypotension occurs due to the non-specific conduction block produced by local anaesthetics, which affects not only the sensory fibers, but also the pre-ganglionic sympathetic fibers resulting into a sympathetic blockade.^8^ This blockade will induce relaxation of blood vessels resulting in venous dilation, and pooling of blood in the venous system. Consequently, venous return to the right side of the heart decreases, ultimately causing a fall in mean arterial blood pressure.^2,9^ In pregnant women, increased sensitivity to local anaesthetics, combined with aorto-caval compression from the gravid uterus that reduces venous return to the heart, further predisposes them to hypotension^10^ Maternal hypotension during caesarean section can adversely affect both mother and foetus, leading to reduced utero-placental perfusion, foetal bradycardia, and acid-base abnormalities, which may compromise neonatal outcomes.^6^

Despite preventive measures such as crystalloid preloading and left uterine displacement, the incidence of hypotension during spinal anaesthesia remains high, highlighting the need for further investigation and optimisation of management strategies.^6,11,12^ Some adjuvants such as midazolam and α−2 agonists have been studied, as they prolong the sensory blockade, and thereby reduce the required dosage of anaesthesia, which may reduce the incidence of hypotension.^13,14^ Globally, hypotension occurs in 50 to 80% of caesarean sections with spinal anaesthesia, with variability largely attributed to differences in study definitions and populations.^15^

Understanding the proportion of pregnant patients who develop hypotension and identifying associated risk factors is crucial for improving maternal care and outcomes. Such knowledge can guide the development of evidence-based guidelines and protocols for the prevention and management of hypotension during spinal anaesthesia in Tanzania. Maternal hypotension reduces uteroplacental blood flow, directly affecting the foetus and leading to acidosis, metabolic derangements and low APGAR scores at delivery.^16–18^ Implementing standardised guidelines will harmonise practices across healthcare facilities and ensure safe administration of anaesthesia for obstetric patients undergoing caesarean section. In Tanzania, there is limited data regarding the proportion of pregnant patients who develop hypotension during spinal anaesthesia and the associated risk factors. This study aims to provide region-specific estimates and close this gap.

## METHODS

### Study Design

This hospital-based descriptive cross-sectional study was conducted to determine the prevalence of hypotension and identify associated risk factors among pregnant patients undergoing caesarean section under spinal anaesthesia.

### Study Duration

The study was conducted over a 6 months period, from August 2021 to January 2022. This timeframe allowed for the enrolment of adequate number of participants and the collection of sufficient data to achieve the study objectives.

### Study Area

The study was carried out in the Obstetric Theatre of Muhimbili National Hospital, the largest referral hospital in Tanzania. Located in Tanzania, the hospital receives referrals from municipal hospitals and other regions of the country. It provided an appropriate setting for the study due to its high volume of obstetric cases and availability of experienced anaesthetists.

### Study Population

The study population consisted of all patients who underwent elective and emergency caesarean sections under spinal anaesthesia at Muhimbili National Hospital. Consecutive sampling was employed, enrolling all patients who met the inclusion criteria and gave consent over the 6-month study period.

### Inclusion Criteria

Obstetric patients classified as ASA II, scheduled for elective or emergency caesarean sections and consenting to receive spinal anaesthesia were included. This ensured that participants had appropriate health status for spinal anaesthesia and were willing to participate in the study.

### Exclusion Criteria

Patients who received sedation or a combination of anaesthesia modalities, including cases where spinal anaesthesia was converted to general anaesthesia, were excluded. Those on anti-hypertensive treatment or with pregnancy-induced hypertension were also excluded. Additionally, patients with a modified Bromage score of 0, 1, or 2 (indicating incomplete motor block) were not included in the study.

### Sample Size Estimation

The sample size was determined using the formula for estimating proportions. The following parameters were used:

Critical value (Z) for a 5% significance level: 1.96 Estimated prevalence (P) of hypotension in patients undergoing caesarean section under spinal anaesthesia: 80%^12^

Margin of error (E): 5%

Using the formula N = Z^2^ × P × (100 - P)/E^2^, the estimated sample size (N) was calculated as follows:



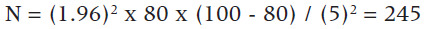



To account for potential missing data, 10% was added to the calculated sample size, yielding a target of 270 patients. Ultimately, 300 patients were enrolled, providing a larger sample size and accounting for potential dropouts and incomplete data.

### Data collection and study procedure

All eligible patients who provided informed consent were included in the study. For elective cases, demographic data was recorded during the pre-visit period, while for emergency cases, data was collected during the pre-anaesthesia evaluation. Pre-operative fasting time was documented prior to surgery. Crystalloid preload was calculated based on patient weight (10–15 mL/kg) prior to spinal anaesthesia; following standardised protocols, even in emergency settings. Baseline blood pressure and heart rate were recorded after patients were connected to an ECG monitor and automated blood pressure machine.

Using aseptic technique, local anaesthesia (2.0% Lidocaine) was administered through skin infiltration, after which spinal anaesthesia was performed in the seated position at the L2–L3 or L3–L4 inter-space using a 25G spinal needle. The intrathecal agent was either 0.5% hyperbaric Bupivacaine or 5% heavy Lidocaine, depending on availability. Following administration, patients were gently assisted into a supine position with left uterine displacement using a wedge to prevent compression of the aorta and vena cava. Examination to confirm adequate block was confirmed and documented before proceeding with caesarean section. Additionally, mothers received 3–5 L/min of supplemental oxygen through a facemask.

The study period commenced at the time of spinal injection and continued for 30 minutes. The upper sensory level of anaesthesia was assessed by evaluating loss of pinprick discrimination, while block height was determined by loss of sensation to cold methylated spirits. Motor block was assessed using the Modified Bromage scale.

Data collection was conducted prospectively using questionnaires completed by the responsible anaesthetist at the end of each operation. Variables such as; age, gravidity, height, preload administration, hypotension and APGAR score were initially recorded as numerical data and later sub grouped into categorical data. Nature of surgery, level of block, type of infusion, size of intravenous cannula, type of anaesthetic agent used and presence of wedge was recorded as categorical data. Hemodynamic variables (Systolic blood pressure, diastolic blood pressure, mean arterial pressure and heart rate) were recorded every 3 minutes using the automated blood pressure monitor, which also stored additional patient records throughout the procedure, including oxygen saturation. Hypotension was defined as either a mean arterial pressure of less than 65 mmHg or a 20% reduction from the baseline mean arterial blood pressure (whichever occurs first).^19^

### Quality Control

Before the study commenced, research assistants received training to ensure proficiency in filling out the study questionnaire. Questionnaires were collected twice daily from the obstetric theatre: once in the evening to capture data from the entire day's cases, and again the following morning to include emergency patients recruited overnight. The principal investigator closely supervised the study to ensure accurate documentation and proper record keeping. This meticulous approach aimed to maintain the integrity and quality of the data collected throughout the targeted study period. Data collectors were not blinded, and this is acknowledged as a limitation.

### Data Analysis

Categorical variables were summarised as frequencies and proportions. This information was then tabulated and presented in form of pie charts and tables. The Chi-square test was used to asses statistical significance. All statistical analyses were performed using SPSS version 27.0, with a 5% significance level applied.

### Ethical Approval and Consent to Participate

Ethical approval was obtained from the Ethics Committee of Muhimbili University of Health and Allied Sciences. The committee ensured that participants’’ rights, safety, and welfare were protected in accordance with established ethical principles. Written informed consent was obtained from all participants before enrolment. For emergency cases requiring immediate intervention, verbal consent was obtained and documented in line with ethical standards.

## RESULTS

**FIGURE 1:**
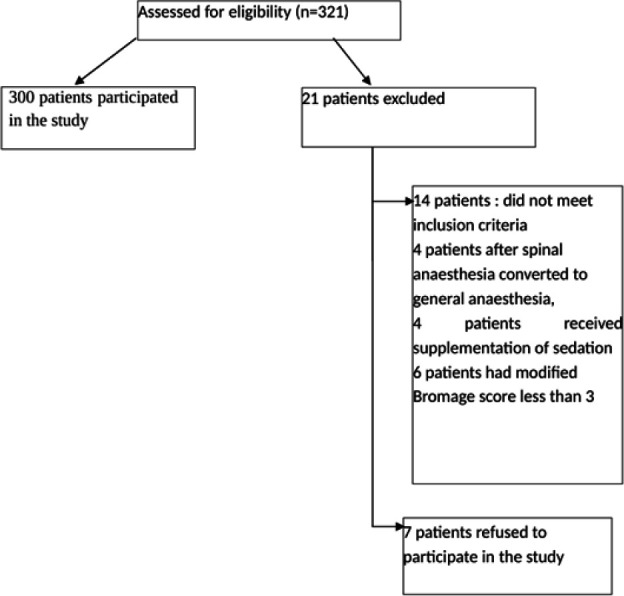
Patient Distribution Flow Chart

A total of 300 pregnant patients comprised the study group (Figure 1), and their demographic data is presented in Table 1. Among the 300 pregnant patients, 33.3% underwent elective caesarean section, while 66.7% underwent emergency caesarean section. The average age of the study population was 29.07 ± 5.5 years, with an average weight of 75.25 ± 13.7 kilograms and an average height of 158.71 ± 9.2 centimetres.

**TABLE 1:** Socio-Demographic Characteristics of Study Participants at MNH 2022 (N = 300)

Variable	Frequency (N)	Percent (%)
Heights (cm)		
≤150	53	17.7
>150	247	82.3
Gravidity		
Primigravida	98	32.7
2–4	182	60.7
>4	20	6.7
Nature of Caesarean Section		
Elective	100	33.3
Emergency	200	66.7
Local Anaesthetics		
0.5% hyperbaric Bupivacaine		
7.5 mg	60	20.0
10.0 mg	207	69.0
12.5 mg	9	3.0
5% heavy Lidocaine		
75 mg	24	8.0
Total	300	100

In the study, 32.7% of the patients were primigravida, while 67.4% were multiparous. Most of the patients (92%) received 0.5% hyperbaric Bupivacaine as the local anaesthetic while 8% received 5% heavy Lidocaine, of which none developed symptoms of transient neurological syndrome. The dosage of 0.5% hyperbaric Bupivacaine varied, with 20% receiving 7.5 mg, 69.0% receiving 10 mg, and 3% receiving 12.5 mg.

**FIGURE 2:**
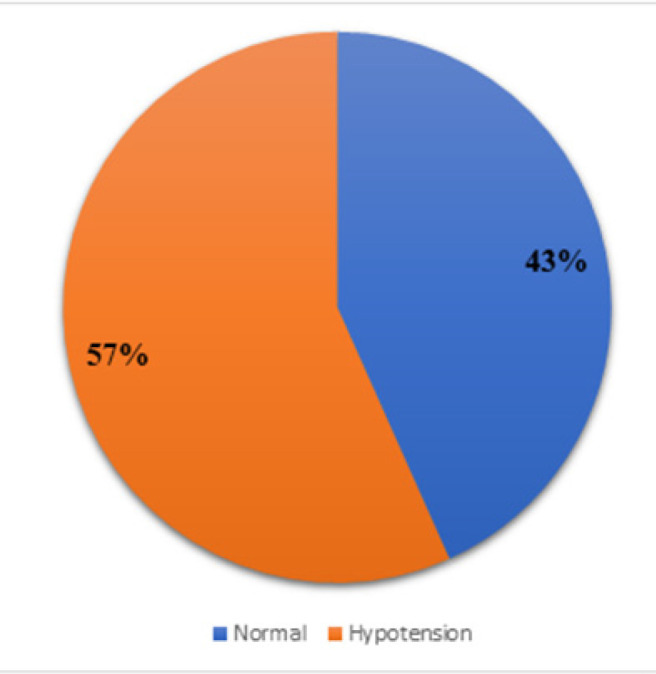
Pie Chart Showing Proportion of Pregnant Patients Who Developed Hypotension

The proportion of pregnant patients who developed hypotension (Figure 2) during caesarean section under spinal anaesthesia was 56.7% (95% CI: 0.511–0.623). However, no association was observed between maternal hypotension and neonatal outcome as assessed by APGAR scores (Table 2).

**TABLE 2:** Distribution of Apgar Score of the Baby Among Pregnant Patients Who Developed Hypotension

Variable	Hypotension. No (%)	Hypotension. Yes (%)	TOTAL (N) (%)	p-value
Apgar score				
Yes ≥ 7	129 (99.2)	164 (96.5)	293 (97.7)	0.117
No < 7	1 (0.8)	6 (3.5)	7 (2.3)	

Factors associated with hypotension are presented in Table 3. A higher proportion of patients (70.2%) with a preload volume below 10 ml/kg developed hypotension. The incidence of hypotension increased with higher sensory block levels (T4–T6). Despite wedge use in 91.7% of pregnant patients, 54.9% still developed hypotension. Hypotension occurred more frequently during emergency caesarean sections e(60.5%) than elective caesarean sections (49%). No significant associations were found with age, gravidity, or choice of local anaesthetic.

**TABLE 3:** Risk factors for development of hypotension for pregnant patient receiving spinal anaesthesia

Factor	Hypotension. No (%)	Hypotension. Yes (%)	TOTAL N (%)	p-value
Preload (ml/Kg)				
Less than 10	17 (29.8)	40 (70.2)	57 (19.0)	.009
10 to 14	54 (42.2)	74 (57.8)	128 (42.7)	
15 to 19	29 (43.3)	38 (56.7)	67 (22.3)	
20 and above	30 (62.5)	18 (37.5)	48 (16.0)	
Level of block				
T4	9 (21.4)	33 (78.6)	42 (14)	.003
T5	63 (41.7)	88 (58.3)	151 (50.3)	
T6	31 (52.5)	28 (47.5)	59 (19.7)	
T7	27 (56.2)	21 (43.8)	48 (16)	
Presence of wedge				
Forgotten	6 (24)	19 (76)	25 (8.3)	.042
Wedge 15° left lateral	124 (45.1)	151 (54.9)	275 (91.7)	
Nature of surgery				
Elective	51 (51)	49 (49)	100 (33.3)	.058
Emergency	79 (39.5)	121 (60.5)	200 (66.7)	
Gravidity				
Prime gravida	47 (36.2)	51 (30.0)	98 (32.7)	.495
1 to 4	74 (56.9)	108 (63.5)	182 (60.7)	
More than 4	9 (6.9)	11 (6.5)	20 (6.7)	
Age				
16 to 19	10 (7.7)	8 (4.7)	18 (6)	.288
20 to 24	15 (11.5)	30 (17.6)	45 (15)	
25 to 29	46 (35.4)	54 (31.8)	100 (33.3)	
30 to 34	38 (29.2)	41 (24.1)	79 (26.3)	
35 to 39	20 (15.4)	32 (18.8)	52 (17.3)	
More than 39	1 (8)	5 (2.9)	6 (2)	
ASA				
I	62 (47.7)	62 (36.5)	124 (41.3)	.348
II	68 (52.3)	108 (63.5)	176 (58.7)	
Local Aesthetics				
5% heavy Lidocaine	11 (46)	13 (54)	24 (8)	.623
0.5% hyperbaric Bupivacaine.	119 (43).	57 (57).	276 (91.7)	

## DISCUSSION

This study highlights the prevalence and associated factors of hypotension in pregnant patients undergoing caesarean section under spinal anaesthesia. The incidence of hypotension was 56.7%, which aligns with findings from studies conducted outside the African subcontinent.^16,19^ Studies in East Africa showed a prevalence range from 30% to 60%.^20–22^ Hypotension remains a well-known complication of spinal anaesthesia, and its occurrence can have significant implications for both the mother and the foetus.^4^

One of the factors that contribute to hypotension during spinal anaesthesia is the sympathetic block induced by local anaesthetics. This block affects not only the sensory fibers but also the pre-ganglionic fibers to the sympathetic chain, resulting in arterial and venous dilatation. The decrease in venous return to the right side of the heart leads to a fall in mean arterial blood pressure. Additionally, the pressure of the gravid uterus on the aorta and inferior vena cava exacerbates the decrease in venous return, further contributing to maternal hypotension.^2,7^ In this study, patients with a preload volume below 10 ml/kg and those with higher sensory block levels were more likely to develop hypotension, underscoring the importance of optimising preload and monitoring block height as preventive strategies.

The prevalence observed in this study was slightly lower compared to some studies conducted in India and Kenya.^9,21^ These differences could be attributed to variations in patient populations, anaesthetic techniques, and clinical management protocols. It is worth noting that the use of different local anaesthetics, such as 5% heavy Lidocaine and 0.5% hyperbaric Bupivacaine, can also influence the incidence of hypotension. In this study, hypotension occurred more frequently in patients receiving Bupivacaine compared to Lidocaine.

Preventive measures, including crystalloid preloading and left uterine displacement with a wedge, were implemented in this study. Although left uterine tilt has been shown to provide modest hemodynamic benefits^22^, more than half of the patients still developed hypotension. This highlights the persistent challenge of preventing maternal hypotension during spinal anaesthesia. Alternative approaches and interventions such as prophylactic vasopressors and perioperative echo graphic assessment may help to reduce the prevalence of hypotension and its associated complications.^21,23^

The level of sensory block was also a significant factor. Patients with a block height at or above T5 experienced more frequent hypotension than those with lower block levels, consistent with previous studies.^1,4^ This reinforces the need for vigilant monitoring of block level to identify high-risk patients and implement timely interventions.

## CONCLUSION

Hypotension remains a common complication of spinal anaesthesia in pregnant patients undergoing caesarean section. This study contributes to existing evidence by identifying preload volume and block height as significant factors associated with its development. Preventive measures such as adequate fluid preload and left uterine tilt, together with careful selection of anaesthetic agents and monitoring of block level, are essential to reduce the incidence and severity of hypotension and improve maternal and foetal outcomes. Future research should focus on optimising anaesthetic protocols and evaluating additional strategies, including prophylactic vasopressors, to enhance the safety and effectiveness of spinal anaesthesia in obstetric patients.

### Limitation

This study has several limitations. First, it was conducted exclusively among healthy ASA II pregnant patients, therefore, the findings may not be generalizable to higher-risk populations. Future research should include women with higher ASA classifications and a broader range of patient profiles to better assess responses to spinal anaesthesia.

Second, some emergency patients may have received aggressive fluid therapy prior to arriving in the operating theatre. As preload measurements were taken only after arrival, prior fluid management may not have fully accounted for, potentially affecting the observed incidence of hypotension.

Finally, this study could not determine whether the choice of anaesthetic agent influenced the development of hypotension. Comparative studies assessing different anaesthetic agents are needed to clarify their relative impact on maternal hemodynamic outcomes.
